# Serendipity and Logic in Medical Research

**Published:** 1991-12

**Authors:** Brandon Lush


### Serendipity and Logic in Medical Research

**Brandon Lush, MD, FRCP**

There are several approaches to medical research — induction, deduction, hypothesis and experiment, etc. — but I shall concentrate on only two in this short talk. Namely, serendipity and logic, with two examples of each. By serendipity I mean the fortuitous turning up of a pleasant and unexpected happening which is recognised as such.

As a member of the staff of the Medical Research Council from 1951 to 1972 it was my good fortune to meet most of the leading medical research workers of that period. Some of their published papers do not give all the details of what led to their results — so herewith an attempt to complete the record.

The late Sir Christopher Andrewes, regarded by many as the founding father of virology research, spent years trying to isolate the influenza virus, which he was convinced must exist — so he sought hard and long for an animal host in which it could be studied. In his own words:

"The epidemic of influenza at the beginning of 1933 afforded an opportunity of making an experimental study of this disease . . . Throat washings were obtained from a number of patients as early as possible after the onset of definite symptoms. On the assumption that the aetiological agent was probably a filterable virus the throat washings were filtered through a membrane impermeable to bacteria. The filtrates, proved to be bacteriologically sterile, were used in attempts to infect many different species. All such attempts were entirely unsuccessful until the ferret was used . . .".

Had a ferret not sneezed when Sir Christopher was by himself in the animal house, he would have given up, because he had previously used every known laboratory animal. It was sheer serendipity that he heard the sneeze and appreciated its significance.

In parenthesis, it is worth noting that such extreme species specificity with human infections is common. R. J. W. Rees and his colleagues proceeded as far as the armadillo before they could find an animal model for leprosy.

Sir Christopher persuaded the MRC to set up a Common Cold Research Unit to try to find the causative virus or viruses — but in ten years he was unable to succeed. He trained a young colleague, D. A. J. Tyrrell, whom the MRC appointed as Director when Sir Christopher retired. David Tyrrell went back to first principles and tried putting up roller-tube cultures at 33°C (since the anterior nares are always colder than the body core temperature) instead of the usual 37°C. At first he succeeded — but he was unable to repeat the result. Being a very logical man he was convinced that there must have been a reason for the differing results — so he cross-examined his team and a technician confessed to making the successful roller-tube too alkaline. Hence, by making the culture ecologically agreeable to the viruses causing the common cold, many of which were subsequently isolated using the same technique.[3,4,5]

Another elegant example of logic was shown in a series of experiments[6] by the late John Squire, who had the distinction of being Director of two separate MRC Units (Industrial Medicine and Experimental Pathology of the Skin) as well as being Head of the Department of Pathology in Birmingham University Medical School. He was sure that the skin oils did more than render skin waterproof. So he tested the survival of

Staph. albus on normal skin and on skin from which the skinoils had been removed by ether — and showed much longer survival in the latter case. He then removed the ether and tested the remaining oils against cultures of Staph. albus — and found that the oils were bactericidal. This led to changes in the approaches of surgeons to skin sterilisation prior to surgery.

Finally, another example of serendipity. Dr. Audrey Smith, Head of the Cryobiology Division of the National Institute for Medical Research, had, over a number of years succeeded in freezing small animals solid and then restoring them to apparently normal life. When she was demonstating this to an official MRC visiting party led by its then Chairman, the late Earl of Limerick, she invited him to feel a lifeless frozen rat. He rubbed one of its ears between his fingers. When it was restored to life it was entirely normal apart from that ear, which was frost-bitten. This chance observation led to the abandonment of the previous policy of massaging frost-bitten extremities with snow.[7] It is far better to wrap them in insulating material (e.g. cotton wool) and to avoid all trauma until revascularisation has occured.

**REFERENCES — see p.118**

**ctd. from p.107 ''Serendipity'' — Lush**

**REFERENCES**

1. The Clinical Evaluation of Remedies, F.H.K. Green, *Lancet.* 1954. **2:** 1085. (The Bradshaw Lecture of the RCP, reprinted in Concepts of Medicine, ed. Brandon Lush, Pergamon Press, 1961).
2. A Virus obtained from Influenza Patients, Smith W.; Andrewes C.H.; Laidlaw P.P.; *Lancet.* 1933. **2:** 66.
3. Laboratory Studies of APC and Influenza Viruses, Balducci D.; Zaiman E.; Tyrrell D.A.J. *Brit.J.Exp.Path.* 1956. **37:** 205.
4. Some Virus isolations from Common Colds. Tyrrell D.A.J.; Bynoe M.L.; Hitchcock G. *Lancet.* 1960. **1:** 235.
5. The cultivation in human embryo cells of a virus causing the common cold in man. Tyrrell D.A.J.: Buckland F.E.: Bynoe M.L.; Hayflick G. *Lancet.* 1962. **2:** 320.
6. Current Therapeutics: XLI Skin Disinfection. Squire J.R. *The Practioner.* 1951. **166:** 502.
7. Viability of supercooled and frozen mammals. Smith A.U.; Ann N.Y. *Acad. Sci.* 1959. **80:** 291.

